# Systematic Study of the Sensory Quality, Metabolomics, and Microbial Community of Fresh-Cut Watermelon Provides New Clues for Its Quality Control and Preservation

**DOI:** 10.3390/foods11213423

**Published:** 2022-10-28

**Authors:** Yili Hu, Yi Cai, Haibin Wang, Yin Xiong, Xinyu Zhang, Liying Wei, Zhixian Qiao

**Affiliations:** 1 National R&D Center for Se-Rich Agricultural Products Processing, School of Modern Industry for Selenium Science and Engineering, Wuhan Polytechnic University, Wuhan 430023, China; 2 Hubei Key Laboratory for Processing and Transformation of Agricultural Products, School of Food Science and Engineering, Wuhan Polytechnic University, Wuhan 430023, China; 3 Tianjin Institute of Industrial Biotechnology, Chinese Academy of Sciences, Tianjin 300308, China; 4 Institute of Hydrobiology, Chinese Academy of Sciences, Wuhan 430072, China

**Keywords:** fresh-cut watermelon, quality changes, sensory parameters, NMR-based metabolomics, microbial community

## Abstract

As a popular form of fruit consumption, fresh-cut watermelon is of great convenience for its consumers. Owing to the lack of comprehensive knowledge about the quality changes of fresh-cut watermelon during its shelf life, guidelines and standards are unavailable currently. To clarify the deterioration process and its underlying mechanism in fresh-cut watermelon, the sensory parameters, metabolomics, and microbial community of fresh-cut watermelon during a three-day storage at both room temperature (RT) and refrigerator temperature were systematically studied in this work. Results revealed that the whole property of the watermelon stored at refrigerator temperature kept stable, while pulps stored at RT had substantially deteriorated after 36 h. The decay was reflected in the significant decrease in soluble solid contents, firmness, pH, and color parameters in the sensory perspective. At the metabolic level, significantly declined malate, citrate, uridine, uridine 5-monophosphate, and amino acids, and increased ethanol and lactate contents, were observed as deterioration markers, which partially resulted from the activities of pyruvate dehydrogenase and alcohol dehydrogenase and the burst of genera *Enterobacteriaceae* and *Leuconostocaceae*. This study unveiled the underlying mechanisms of quality changes in fresh-cut watermelon under its primary storage conditions to provide fundamental information and potential clues for its quality control and preservation.

## 1. Introduction

Watermelon (*Citrullus lanatus*) is a popular fruit because of its fresh and juicy pulp. Compared with other fruits, watermelon can hardly be finished at once due to its relatively large volume. Therefore, the pervasive practices for watermelon consumption are slices and cubes in both markets and homes. However, the exposed pulps inevitably suffer from texture breakdown and microbial contamination, leading to fast deterioration. Alimentary toxicosis incidents that result from eating questionable watermelons occur occasionally every summer. Meanwhile, few guidelines and standards specially for fresh-cut fruits and vegetables have been introduced. To improve the quality control and preservation of fresh-cut watermelon, the metabolic processes, including enzymatic reaction, ethylene production, and respiration, and the impacts from microbials, need to be illustrated.

Previous studies on watermelon mainly focused on exploring feasible techniques that could extend its shelf life [1,2,3,4]. Optimum modified atmosphere packaging in higher than 14% O_2_ atmosphere was reported to extend the shelf life of fresh-cut watermelon, while visible light exposure of 3000 Lux was reported to decrease drip loss of watermelon by accelerating moisture evaporation and reducing cell wall degradation and the activity of polygalacturonase [3]. UV-C illumination is another effective pretreatment in the fresh-cut industry. The shelf life of fresh-cut watermelon cylinders was extended to 7 days after the application of 4.8-kJ m^−2^ UV-C dose [1]. To evaluate the quality of watermelon pulps during storage, numerous property indices have been employed in previous research, including respiration rate, microbial quality, color, weight loss, and enzyme activity. The quality changes in watermelon are mainly attributed to autologous metabolism and microbial activities. Nevertheless, not much is known about the changes in global nutritional components and involved microbial community in watermelon during its shelf life.

Aside from macroscopic properties, metabolomics and microbial ecology are other powerful tools to unveil the biological process and microbial coaction during food storage. Metabolomics has been applied to detect nutritional differences in fruits, meat, and other processed foods in food science [5]. The metabolic profiles of strawberry with different edible coatings [6], fresh-cut browning in apple [7], tomato at different maturation levels [8], and chilled chicken meat at different storage times [9] have been explored with the aid of metabolomics. Similarly, a comparative metabolomics profiling conducted on fresh-cut melon (cv. Xizhoumi-17) has revealed 17 differential metabolites during storage at 15 °C compared to totally fresh melon, among which salicylic acid beta-D-glucoside, the cut-wounding stress indicator, was observed to be increased significantly [10]. The accumulation of pyruvate-derived metabolites (ethanol, acetaldehyde, lactate, alanine) in apple during its shelf life was uncovered by an integrated metabolomics approach. It has been hypothesized as being a hint of a metabolic reconfiguration strategy in apples to regenerate NAD+ and cope with energy crisis under hypoxia [11]. Such information provides an important theoretical basis to explain the mechanisms underlying changes in food quality, which benefits the improvement of food storage technology.

As another vital factor that affects food quality during storage, microbial activity is usually evaluated in accordance with national standards in spite of their deficiency in unraveling the microbial communities from different food matrices. Benefiting from the revolutionizing development of next-generation sequencing, studies on microbial ecology have become popular in food science [12]. However, most studies are devoted to the analysis of microbial communities in fermented foods to achieve better manipulation of fermentation microorganisms and improve food production, whereas research on microbial ecology concerning food quality and preservation is relatively inadequate.

To clarify the deterioration process and its underlying mechanism in fresh-cut watermelon during daily storage, as well as explore the boundary for its quality control, individual watermelon cubes packed with films and stored under constant-temperature conditions were monitored. The temperatures of 28 °C and 4 °C were chosen in this work due to their representativeness for room temperature (RT) in the watermelon-consuming season and for refrigerator atmosphere, respectively. Fresh-cut watermelon cubes were sampled at eight successive storage time points (0, 3, 6, 12, 24, 36, 48, and 72 h) and subjected to studies of physicochemical properties, metabolomics, and microbial community by using conventional and advanced techniques. This study established a new platform for evaluating the overall quality of fresh-cut watermelons. This platform is beneficial for unveiling the underlying mechanism of quality changes in fresh-cut watermelon under its primary storage conditions to provide fundamental information and potential clues for its quality control and preservation.

## 2. Materials and Methods

### 2.1. Materials and Sample Preparation

Watermelons were purchased from the Wushang Supermarket in Wuhan, China. Careful inspection was conducted to guarantee no visual defects in these melons, and melons with uniform shape and size were selected. Then, the watermelons were rinsed with double-distilled water and divided into cubes (approximately 3 cm × 3 cm × 3 cm). The knife and containers were sanitized with 75% ethanol before use. Regular polyethylene (PE) wraps were used to pack fresh-cut watermelons. Half of the samples were kept at refrigerator temperature (4 °C), while the other half were stored at RT (28 °C). Fresh-cut melons that had been stored for several hours (3, 6, 12, 24, 36, 48, and 72 h) were sampled randomly and subjected subsequently to the experimental studies listed below.

### 2.2. Physicochemical Properties

#### 2.2.1. Microscopic Examination

An inverted microscope (Nikon Eclipse TS100, Nikon Instruments Inc., Melville, NY, USA) was employed to capture the images of fresh-cut watermelon at a series of successive storage times. Small pieces were excised with a cork borer (#7, 4 mm diameter), and a transversal cut was conducted using a stainless steel blade. Sample surfaces of watermelon pieces were observed with a 40 magnification lens, and the images were recorded with NIS-Elements BR 3.2 software (Nikon Instruments Inc., Melville, NY, USA).

#### 2.2.2. Color and Firmness Measurements

Flesh color (CIE L*, a*, b*) was measured using a chroma meter (CR-400, Konica Minolta, Tokyo, Japan) calibrated with a white tile. Color indices were expressed as lightness L*, chromaticity a* and b*.

Penetration tests were performed using a texture analyzer (TA-XT2 Plus, Stable Micro Systems, Godalming, UK). The sample was placed on the plate before a cylindrical probe (5 mm diameter) was pushed into the watermelon at a speed of 1.5 mm/s.

#### 2.2.3. Soluble Solid Contents (SSCs) and pH Measurements

The watermelon samples were homogenized using a blender (JYZ-D02V, Joyoung, Jinan, China). SSCs of watermelon cubes were analyzed using a refractometer (model PR-101, Atago Co., Tokyo, Japan). The pH of the homogenized watermelon juice was measured using a D-72 pH meter (FE28, Mettler Toledo, Zurich, Switzerland).

#### 2.2.4. Water Contents

An amount of 10 g of watermelon was lyophilized with a freezer dryer (SCIENTZ-18N, SCIENTZ, Ningbo, China). Accurate weights before and after lyophilization were recorded and employed in water content calculation.

### 2.3. Biochemical Properties

#### 2.3.1. Protein Contents

The Plant Total Protein Extraction Kit (APPLYGEN, Beijing, China) was employed in accordance with the instructions. Four hundred milligrams of watermelon pulps was suspended in 1.0 mL of an extraction solution and homogenated completely. After being incubated on ice for 20 min, samples were centrifugated at 12,000× *g* for 15 min, and supernatants were collected for protein quantitation. The protein content of each sample was measured using the Bradford method with bovine serum albumin as standard.

#### 2.3.2. Nuclear Magnetic Resonance (NMR)-Based Metabolomic Analysis

Sample preparation for NMR analysis was carried out as previously described [12] with some modifications. Watermelon samples were extracted thrice with 1 mL of a methanol/water mix [2:1 (*v*/*v*)], followed by evaporation with a SpeedVac vacuum concentrator (Thermo Fisher Scientific, Waltham, MA, USA). Extracts were suspended in 600 μL of PBS [0.1 M, 50% (*v*/*v*) D_2_O, and 0.005% 3-(trimethylsilyl)[2,2,3,3-D4] propionate] and centrifugated at 12,000 g for 10 min. Supernatants were transferred to NMR tubes and analyzed with a Bruker Avance III 800 MHz NMR spectrometer equipped with an inverse cryogenic probe (Bruker Biospin, Rheinstetten, Germany). A typical one-dimensional hydrogen NMR spectrum was acquired using the first increment of nuclear Overhauser effect spectroscopy sequence with presaturation. For signal assignment, ^1^H-^1^H correlation spectroscopy (COSY), ^1^H-^1^H total correlation spectroscopy (TOCSY), ^1^H J-resolved spectroscopy (J-Res), ^1^H-^13^C heteronuclear single quantum correlation spectroscopy (HSQC), and ^1^H-^13^C heteronuclear multiple bond correlation spectroscopy (HMBC) were performed as described previously [13].

The ^1^H spectral region of δ 0.5–9.5 was integrated into bins by using AMIX package (v3.8, BrukerBiospin). Each bin area was normalized to the weight of pulps. Principal component analysis, and orthogonal projection to latent structure-discriminant analysis (OPLS-DA), were performed with SIMCA-P+ software (v 14.0, Umetrics, Umeå, Sweden).

The models were built using OPLS-DA with Pareto variance (Par) scaling and evaluated by sevenfold cross-validation and the cross-validated residuals (CV-ANOVA). To assist the biological interpretation of information generated from these models, the loading values were back-transformed and plotted with color-coded OPLS-DA coefficients by using MATLAB (v 9.9, MathWorks, Natick, MA, USA).

### 2.4. Microbial Analysis

#### 2.4.1. Microbial Population Assay

At each sampling time, watermelon cubes were randomly selected and blended into juice. Subsequently, 1 mL of the juice was added into a sterilized tube containing 9 mL of 0.85% sterile saline for serial dilution. Then, 0.1 mL of the dilution was spread on a trypticase soy agar (T8650, Solarbio, Beijing, China) culture medium in duplicate and incubated at 30 °C for 24 h. Finally, the colony numbers on the culture dish were counted and calculated as log10 colony forming units (CFU) per gram. The results were expressed as mean values for three individual watermelon cube samples (*n* = 3).

#### 2.4.2. 16S rRNA Gene Sequencing and Analysis

The TIANGEN kit (TIANGEN Biotech, Beijing, China) was used to extract genomic DNA from the microbial community in watermelon pulps. DNA concentrations were quantified using a Qubit 4.0 fluorometer (Q33226, Thermo Fisher Scientific, Waltham, USA) and normalized to a concentration at 10 ng/μL. The V3–V4 region (~450 bp) of the prokaryotic microbial 16S rRNA was amplified with the forward primer 341F (ACTCCTACGGGRSGCAGCAG) and reverse primer 806R (GGACTACVVGGGTATCTAATC) [14] in a PTC-100 thermal controller (MJ Research Inc., Waltham, MA, USA). Nontemplate controls were used as negative controls for each set of barcoded primers. The presence of amplicons was confirmed by gel electrophoresis on a 2% agarose gel and staining with SYBRGreen (TIANGEN Biotech, Beijing, China). Equimolar amounts of the PCR amplicons were mixed in a single tube, and amplification primers and reaction buffer were removed by processing the mixture with the Agencourt AMPure XP Kit (Beckman Coulter Genomics, Danvers, MA, USA). The purified amplicons were sequenced using MiSeq (Illumina, CA, USA).

Bioinformatic analysis was conducted in USEARCH 11.0.667 and QIIME 2. Reads were filtered before being clustered into operational taxonomic units (OTUs) with 97% sequence identity [15]. Alpha diversity was evaluated using Simpson, Shannon, and Chao1 indices. Beta diversity was estimated by quantifying the degree of dissimilarities in microbiome composition among sites by principal coordinate analysis (PCoA), in which a weighted UniFrac distance matrix was employed to account for OTU abundance and phylogenetic ancestry [16]. Spearman correlation matrices and heat maps were constructed using R version 4.0.5.

### 2.5. Statistical Analysis

Results were expressed as mean ± standard deviation of at least triplicate measurements. One-way ANOVA, Student’s *t* test, or Mann–Whitney U-test was performed to determine the significance between groups at 95% confidence interval via IBM SPSS Statistics (v 26.0, IBM, New York, NY, USA).

## 3. Results and Discussion

### 3.1. Physicochemical Changes in Fresh-Cut Watermelon during Storage

**Figure 1:**
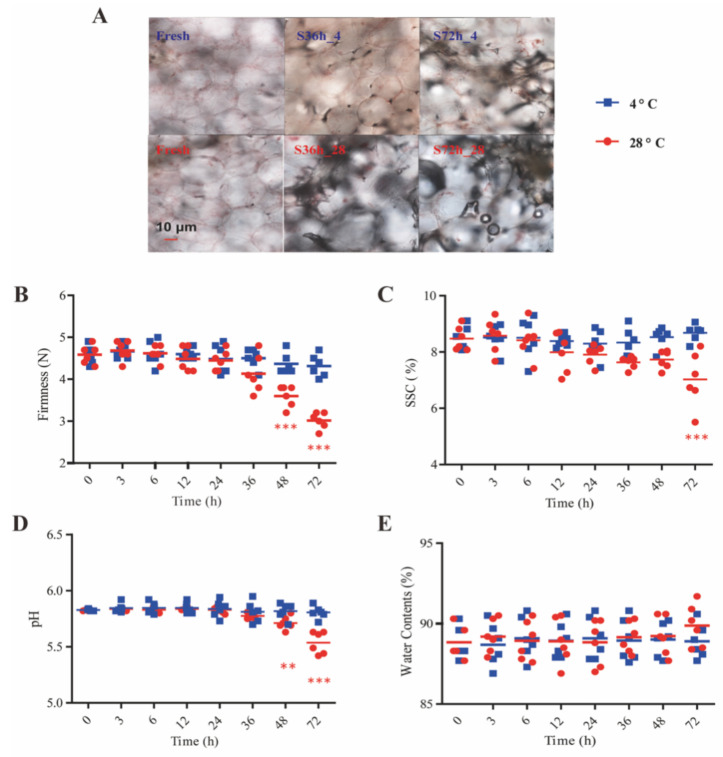
Changes in sensory qualities of watermelon pulps at different storage conditions: (**A**) cell morphology of pulps under microscope; (**B**) firmness; (**C**) soluble solid contents; (**D**) pH; (**E**) percentages of water contents in fresh-cut watermelons. Note: Star in red color means significant difference between the fresh cubes and cubes stored at 28 °C (**p < 0.01, ***p < 0.001 vs fresh group).

The general property of watermelon pulps could usually be assessed with quality parameters, such as firmness, SSC, pH, weight loss, color, and overall acceptability [1,2]. From these parameters (Figure 1, Table S1), storage at a lower temperature effectively slowed down the rate of property deterioration in watermelon, given that no significant changes were captured within 72-h storage at refrigerator temperature. Pictures of watermelon pulps under the microscope (Figure 1A) depicted visual distinctions among pulps in different storage conditions at the micro level. Uniform, well-stacked, and intact appearance of fresh pulp cells was clear, while pulp cells left at RT were in irregular shapes and suffering from precipitation, cell disruption, and microbial contamination simultaneously. Quantizable parameters agreed well with these micrographs.

The average firmness of fresh-cut cubes was 4.62 N, and the value kept almost constant at refrigerator temperature for at least 72 h (Figure 1B). However, the firmness parameter declined after watermelon cubes were stored at RT for more than 2 days. The mechanical strength of parenchyma cell walls, the extension between adjacent pulp cells, and the degree of cell turgor are major determinants of flesh firmness [17]. Generally, the depolymerization of pectin and multiple polysaccharides is regarded as an important factor that results in firmness decrease in most fruits [18,19]. The hydrolysis of pectin and the loss of neutral sugars from polysaccharides weaken cube firmness. Meanwhile, a significant decrease in SSC in fresh-cut cubes was observed after 72 h at RT (Figure 1C).

SSC is used as a marker to predict the sweetness of melon [20] and has been reported to show an uptrend in postharvest fruits [21]. Decreased SSC indicates a reduction in total sugars, which probably results from pulp cell metabolism and microbial activity instead of firmness loss. Similar results were presented in a paper on a study of litchi storage quality under RT [22]. In addition to sugars, the content of acids also exerts a profound impact on fruit flavors. Fresh-cut watermelons kept in a refrigerator maintained their flavors and textural parameters, while cubes stored at RT underwent significant acidity increase when left for more than 48 h (Figure 1D). Along with the reduction in SSC, the rising acidity could probably be attributed to the fermentation of sugars by microorganisms, thus leading to increased production of organic acids and declined pH value [23]. Figure 1E unveils the fact that moisture contents in watermelon stored in both temperatures remained stationary within 3 days, which probably resulted from the contribution of PE wraps and implied that any simultaneous changes in biochemical concentrations in the present work were ascribed to the changes in constituents themselves.

Color parameters monitored during storage are listed in Table S1. The values of L*, a*, and b* imply the lightness, redness, and yellowish degrees of corresponding samples, respectively. The results shared similarity to the changes in the above physiological indices and attested to the phytochemical property variations revealed in watermelon juice during storage [23]. The significantly lower L* and a* values compared with those of fresh cubes suggested a dark and less red appearance. Pigment loss, the Maillard reaction, and polyphenol oxidase account for browning reaction in vegetables and fruits [24,25]. Specifically, the Maillard reaction that occurs between reducing sugars (glucose and fructose) and plentiful amino acids is a crucial factor in pulp browning. Given that enzymatic browning rarely occurs in watermelon flesh, the promoted carotenoid and lycopene degradation and ascorbate oxidation under air exposure at RT were supposed to be the major factors that resulted in color change in this study. Despite the degradation in pulp cell walls and the observed physiological alterations, the water contents of fresh-cut watermelons were constant at around 89.0%, suggesting that no water loss occurred during the three-day storage in these conditions.

### 3.2. Identification and Multivariate Statistical Analysis of Chemical Changes in Fresh-Cut Watermelon during Storage

**Figure 2:**
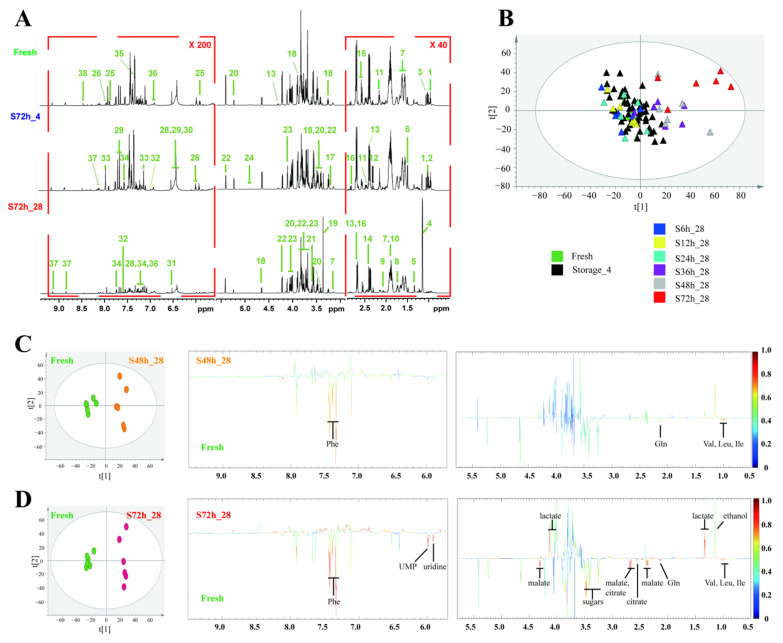
Changes in metabolic profiles of watermelon pulps at different storage conditions: (**A**) 1H NMR spectra of watermelon pulps extracts from fresh cubes and cubes stored for 72 h at 4 °C and 28 °C, respectively; (**B**) PCA score plot for watermelon samples that have been monitored at different storage periods and conditions; (**C**) score plot and loading plot of model constructed using fresh cubes and cubes stored at 28 °C for 48 h; (**D**) score plot and loading plot of model constructed using fresh cubes and cubes stored at 28 °C for 72 h. Note: Identified metabolites are listed in Table S2 with details.

The ^1^H NMR spectra of watermelon flesh extracts are presented in Figure 2A. In accordance with a series of signal information provided by chemical shifts, J-Res, COSY, TOCSY, HSQC, and HMBC, 38 major metabolites in watermelon flesh were assigned and denoted; they included amino acids, sugars, organic acids, and nucleotides, which formed the main material basis of watermelon pulps and covered most phytochemicals reported in previous studies [26,27].

In this study, the metabolic profiles of pulps at different storage states were evaluated to assess the centralized or dispersed distributions among samples. From the reduced dimension feature of flesh extracts shown in Figure 2B, a distinct separation tendency was observed between the flesh extracts of the watermelon cubes stored in a refrigerator within 3 days and the cubes stored at RT for more than 36 h, which was in accordance with the physical and physiological data in Figure 1.

Supervised OPLS-DA models helped quantize group separation parameters and visualize significant differences. Rigorous cross-validations were conducted to calculate the values of Q2 and R2, where Q2 indicates how well a variable could be predicted, and R2 indicates how well the variation in a variable could be explained [28]. As time passed, the ascending Q2 value of models established between fresh pulps and flesh stored at RT clearly manifested the quality shift at the metabolic level (Table 1). Permutation tests (Figure S2) and *p* value of CV-ANOVA further certified the robustness and predictive ability of these models. Multivariate statistical analysis of metabolites from continuously monitored watermelon implied an apparent overall quality decline in flesh after exposure at RT for more than 36 h. Therefore, in consideration of the metabolomic results in this research, watermelon cubes that have been stored at RT for more than 36 h in summer are suggested to be discarded.

**Figure 3:**
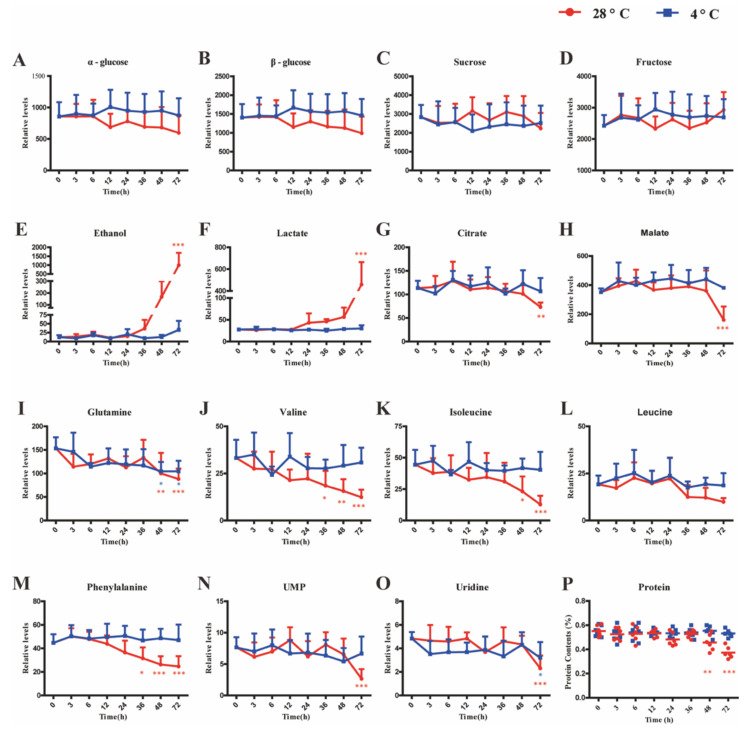
Relative levels of important constitutes and potential biomarkers. Note: Star in red color means significant differences between the fresh cubes and cubes stored at 28 °C, while star in blue color means significant difference between the fresh cubes and cubes stored at 4 °C (* *p* < 0.05, ** *p* < 0.01, *** *p* < 0.001 vs. fresh group).

To determine the constituents that prominently affected the metabolic profiles of multivariate statistical models, the coefficient values of the first principal component in OPLS-DA were obtained for further analysis. As depicted in Figure 2C,D, glutamine, valine, leucine, isoleucine, and phenylalanine decreased evidently after 48 h storage at RT, and the tendency became more remarkable at 72 h. In addition, the contents of malate, citrate, uridine, and uridine 5-monophosphate (UMP) declined, while ethanol and lactate increased significantly, after 72 h storage at RT. Relative quantifications of the above components and soluble sugars were executed by manually integrating their characteristic signals in ^1^H NMR spectra. From the line charts (Figure 3), the variation trends of corresponding components during the whole storage period are comprehensible at a glance.

Amino acid reduction is common in the storage period of vegetables and fruits [29,30], which probably results from the Maillard reaction or enzymatic metabolisms. To explore the mechanism underlying amino acid (glutamine, valine, leucine, isoleucine, and phenylalanine) loss, the total protein contents of watermelons were measured. The significantly downregulated protein percentage of pulps that had been stored at RT for more than 36 h (Figure 3P) presented a negative contribution to synchronous amino acid reduction. Thus, microbial consumption could possibly be the major reason for protein and amino acid reduction, given that branched-chain amino acids, including valine, leucine, and isoleucine, exhibited the same supporting role in microbial growth when compared with total nutrient solution [31]. The rapid proliferation of microbes might deplete existing nucleotides such as uridine and UMP in watermelon flesh and lead to a significant decrease in uridine and UMP.

Despite a declined SSC value being observed in pulps that had been stored at RT for 72 h (Figure 1C), the contents of α-glucose, β-glucose, fructose, and sucrose were statistically stable within 72 h at the two set storage temperatures (Figure 3). On the one hand, the soaring lactate indicated extremely active anaerobic glycolysis and glucose dissipation in pulp cells [32]. On the other hand, firmness loss might upregulate the concentration of sugars to a greater or lesser degree. The consistent contents of certain carbohydrates were speculated to be results of the balance between glucose metabolism and polysaccharide hydrolysis. SSC and semiquantitation of ^1^H NMR signals both showed limitations in representing the carbohydrate profile.

Owing to the intense lactate production through glycolysis, the tricarboxylic acid (TCA) cycle in pulp cells was deeply affected by the suppressed pyruvic acid supply and elevated respiration rate, with significantly decreased malate and citrate as evidence. A similar phenomenon was reported in harvested kiwifruit during storage at RT [33] and postharvest pear fruit under hypoxia [34], which showed pervasiveness in the storage of fruits and vegetables. Pyruvic acid from glycolysis is the major substrate for ethanol fermentation [35], and a malfunctioned TCA cycle tends to feed the pyruvic acid to ethanol fermentation in plants [36]. In the present work, the exponential growth of ethanol was parallel with the decrease in malate and citrate, indicating that relevant enzymes, such as pyruvate dehydrogenase and alcohol dehydrogenase, played vital roles in pulp deterioration [33,37]. Therefore, targeted inhibition of these enzymes could be a potential path to delay property degeneration in watermelon flesh under RT storage.

### 3.3. Microbial Population and Ecology Analysis of Fresh-Cut Watermelon during Storage

**Figure 4:**
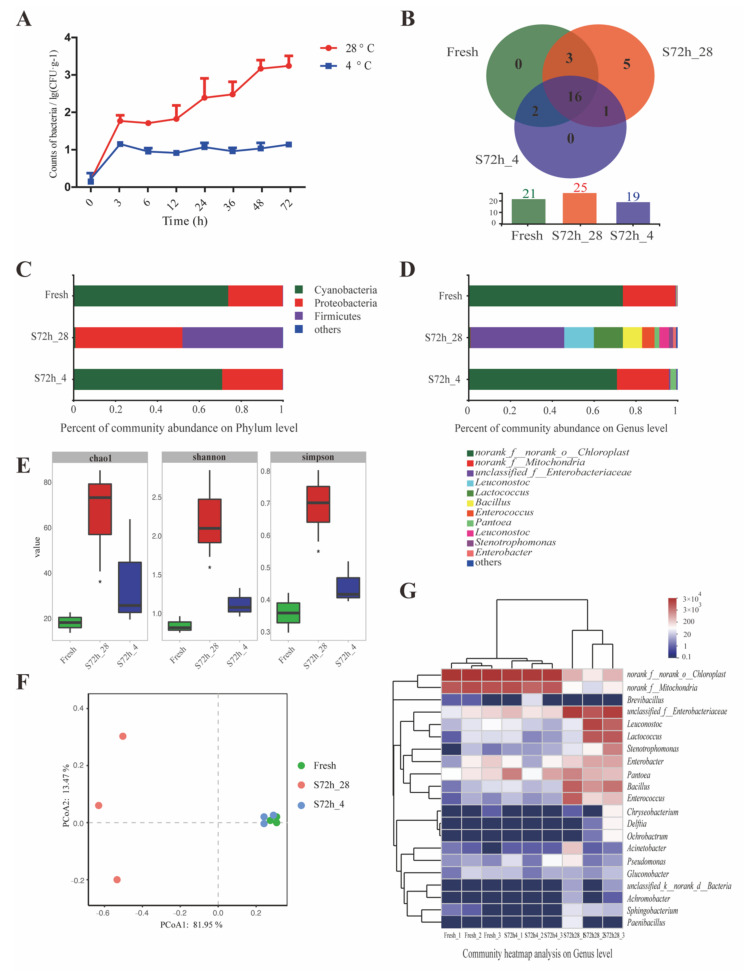
Changes in microbial communities of watermelon pulps at different storage conditions: (**A**) microbial population on watermelon cubes under different storage period and temperature; (**B**) Venn diagram representing the number of shared OTUs between groups Fresh, S72h_4, and S72h_28 using the full data set as well as the unique OTUs of each type of sample; (**C**) relative abundance of microbiota on Phylum level; (**D**) relative abundance of microbiota on Genus level; (**E**) α-diversity indices of microbiome of watermelon cubes from groups Fresh, S72h_4, and S72h_28; (**F**) principal co-ordinates analysis (PCoA) of samples from groups Fresh, S72h_4, and S72h_28; (**G**) community heatmap analysis of samples from groups Fresh, S72h_4, and S72h_28. Note: In figure (**A**,**B**,**E**,**F**), Fresh group is represented in green, S72h_28 group is represented in red, and S72h_4 group is represented in blue; * in figure E means *p* < 0.05 between groups Fresh and S72h_28.

To investigate the general factors that influenced the quality of pulps, both microbial population and ecology were considered in this work. Figure 4A displays the microbial populations in watermelon cubes during the 72 h storage period. An outburst of microorganisms could be noticed after the watermelon cubes were stored for 24 h at RT, while the growth of microorganisms in the refrigerated atmosphere was quite restrained. The initial bacteria count was 0.15 log CFU/g in fresh pulps under a normal dissection circumstance, but this number reached 3.26 log CFU/g after 72 h under RT. According to the current standard system, there is no specific sanitation standard for non-prepackaged ready-to-eat food in China. The demarcation line employed for common foods is 10,000 CFU/g, which is apparently inapplicable in fresh-cut fruits and vegetables. In European Commission No. 1441/2007 on microbiological criteria for foodstuffs, 1000 CFU/g Escherichia coli is set as the upper limit for fresh-cut fruits and vegetables, but APC is not considered. Given that the multivariate statistical analysis of phytochemicals in watermelon demonstrated a significant deterioration resulting from microbial proliferation at 48 h under RT, our APC result at 48 h might be a considerable reference for the upper limit in future standard preparation.

As for the microbial community, DNA extraction of bacteria from fresh flesh and cubes stored for 72 h was further conducted, and the extract was used as a template for the V3–V4 regions of the 16S rRNA gene sequencing. After low-quality sequences were filtered out, 376,612 effective sequences were obtained, with an average length of 456 bp. In spite of the chloroplast and mitochondrial contamination in plant samples [38], chloroplast and mitochondrial units were still retained in our analysis due to the significance of their relative changes.

Based on the sequencing data, a sum of 27 OTUs and 27 species belonging to five phyla were detected in the watermelon samples. The s72h_28 samples had the highest number of species (25 OTUs), followed by the fresh (21 OTUs) and s72h_4 (19 OTUs) samples. The Venn diagram (Figure 4B) revealed five unique OTUs in the s72h_28 samples, whereas no unique OTUs were observed in the fresh and s72h_4 samples.

The alpha and beta diversity analyses are presented in Figure 4E,F, respectively. Satisfactory coverage values were observed in all samples, even though Chao1 diversity indices were considerably higher than the number of OTUs, which indicated that additional bacterial phylotypes were present [39]. Consistent with the previous results, the Chao1, Simpson, and Shannon indices were significantly increased in the s72h_4 samples, indicating that the RT storage induced higher microorganism community diversity. Beta diversity was assessed via the Bray–Curtis hierarchical clustering analysis of watermelon samples, as illustrated in Figure 4F. PCoA suggested significant differences between the fresh watermelon and other batches, with the s72h_28 batch having distinct microbiota compositions that clustered separately from the s72h_4 and fresh groups.

The differences in microbial diversity among the three batches are presented in Figure 4C,D. Cyanobacteria, Proteobacteria, and Firmicutes were the predominant phyla, while *norank_f_norank_o_Chloroplast*, *norank_f_Mitochondria*, and *unclassified_f_Enterobacteriaceae* were the top three genera from our watermelon samples. The similarity of microbial structure between the fresh watermelon and the watermelon stored in a refrigerator revalidated that microbial growth was restrained by low temperature [40].

On account of the distinct transformation of microbial composition at RT, more attention had been paid to the s72h_28 batch. Along with the drastic shrinkage of *norank_f_norank_o_Chloroplast* and *norank_f_Mitochondria* in s72h_28 flesh, the percentage of Firmicutes and Proteobacteria remarkably expanded to 47.88% and 51.10%, respectively (Figure 4C). At the genus level, *unclassified_f__Enterobacteriaceae* (44.76%), *Leuconostoc* (18.83%), *Lactococcus* (13.90%), *Bacillus* (9.17%), *Enterococcus* (5.95%), *Pantoea* (2.39%), *Stenotrophomonas* (1.90%), and *Enterobacter* (1.41%) exhibited an amount advantage over the two other batches (Figure 4D), among which *Leuconostoc*, *Lactococcus*, and *Enterococcus* are typical lactic acid bacteria (LAB). These results showed consistency with the Bray–Curtis hierarchical clustering analysis in Figure 4F. In the identified genera, decreased *norank_f_norank_o_Chloroplast* and *norank_f_Mitochondria* levels and elevated levels of *unclassified_f_Enterobacteriaceae* and other genera that were mainly from Firmicutes and Proteobacteria phyla were revealed to be the major discrepancies between s72h_28 flesh and the two other groups (Figure 4G). As two overwhelming compositions in fresh watermelon and s72h_4 flesh, *norank_f_norank_o_Chloroplast* and *norank_f_Mitochondria* represented chromoplasts and mitochondria, respectively, in consideration of their similarities in 16S sequences [41,42]. The collapse of chromoplasts in pulps of the s72h_28 batch might be responsible for the color change to a certain extent, given that the stability of chromoplasts under different storage conditions is regarded as an important factor that affects pigment degradation rates and color attributes [43]. Moreover, the integrity loss of mitochondria indicated dysfunction of the TCA cycle in melon cells’ metabolism, which had been demonstrated earlier in this article.

Microorganisms play vital roles in food spoilage [44], and food scientists have devoted considerable efforts to identify specific spoilage organisms (SSOs) in various foods [45]. Given microbes of rich numbers in the s72h_28 batch, families of *Enterobacteriaceae*, *Leuconostocaceae*, *Streptococcaceae*, and *Erwiniaceae* were commonly reported to be dominant spoilage bacteria in different food categories, such as peeled potatoes [46] and sliced chicken that had been stored at 25 °C [40], which could be attributed to their preponderance in colonization and proliferation.

### 3.4. Correlation Analysis of Chemical Changes and Microbial Community in Fresh-Cut Watermelon

Trials of targeted inhibition on SSO showed a great potential for application in food preservation. To explore specific bacteria that were responsible for the metabolic profile variations and quality deterioration of the watermelon stored at RT, the Spearman correlation analysis was performed to evaluate the potential correlations between significantly changed metabolites and microbial genera.

**Figure 5:**
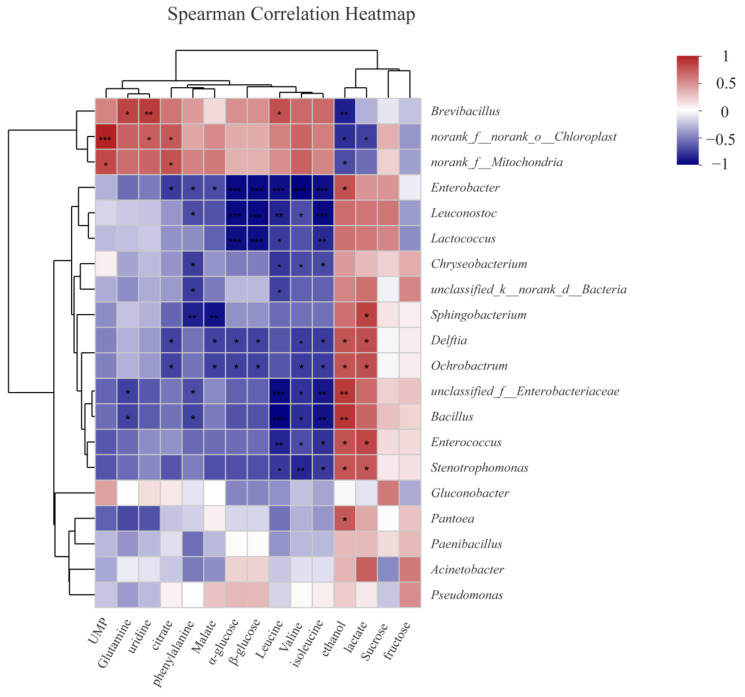
Heatmap of Spearman correlation analysis between the genera of microbiome in fresh-cut watermelons and the significantly changed metabolites in watermelon pulps. Note: Color in red means positive correlation between the genus and corresponding metabolite while blue means a negative correlation relationship; significance of correlation coefficient is denoted in star (* *p* < 0.05, ** *p* < 0.01, *** *p* < 0.001).

As shown in Figure 5, the genera of *Enterobacter*, *Leuconostoc*, and *Lactococcus* were negatively associated with the amount of α-glucose and β-glucose, suggesting a significantly close relationship between these genera and the decrease in glucose, which probably implied a preferable glucose consumption in these genera. Glucose was demonstrated to be the most effective carbon source for *Leuconostoc gelidum* subsp. *gasicomitatum*, a specific strain from the genus *Leuconostoc* [47]. Cell growth was markedly improved when glucose was utilized as the carbon source in a genus, *Lactococcus* [48]. Given that the genera of *Enterobacter*, *Leuconostoc*, and *Lactococcus* occupied only 34.14% of the microbial population in total, with a certain amount of glucose offset from polysaccharide disruption, the levels of both α-glucose and β-glucose were statistically stable in the present study. Even though glucose is recognized as one of the major carbon sources in microorganism growth and reproduction, it is not preferable for the entire population. For some bacteria in tuna, amino acids were reported to act as the preferable carbon sources instead of glucose [49]. For the genera that accounted for more than 1% of the microbial percentage, the levels of dominating amino acids were revealed to have a negative correlation with them (Figure 5), indicating that amino acids, such as Leu, Val, Ile, and Phe, were the preferred carbon and nitrogen sources for these microorganisms.

As an important marker of watermelon deterioration, the drastic increase in ethanol was found to be positively interrelated with *Enterobacteriaceae*, *Bacillus*, *Enterococcus*, *Pantoea*, *Stenotrophomonas*, and *Enterobacter* and was negatively associated with *Brevibacillus*. Previous studies have implied that *Enterobacteriaceae* and some LAB play a role in spoilage fermentations [50,51], while *Pantoea*, *Bacillus*, and *Enterobacter* were identified as parts of the major genera in alcoholic fermentation [52,53]. From the above hints, the elevation of ethanol was a result of spoilage fermentation induced by these microorganisms, and the inhibition of these very microorganisms could be a noteworthy clue for preservation of fresh-cut watermelons. Contrary to microbes (including *Enterobacteriaceae*), *Brevibacillus*, widespread Gram-positive bacteria recorded from diverse environmental habitats, showed a negative correlation with ethanol change in deterioration. Plenty of strains of *Brevibacillus* have been found to produce a biologically active substance with antibacterial and antifungal effects, and species that are currently listed under the genus *Brevibacillus* (formerly, Bacillus brevis cluster) have been a rich source of antimicrobial peptides for many decades [54,55]. The negative correlation of *Brevibacillus* with ethanol change in watermelon deterioration could probably be attributed to its antibiotic secretion potential. Nonetheless, the antagonism from the genus *Brevibacillus* was still limited because of its population disadvantage in the present case.

## 4. Conclusions

In this work, the sensory quality parameters, metabolomics, and microbial community of fresh-cut watermelon pulps during a three-day storage period in RT and refrigerator temperature conditions were systematically studied. Comprehensive data supported that cubes stored at RT for more than 36 h exhibited significant quality deterioration in sensory, metabolic, and microbial perspectives, while fresh-cut watermelons stored at refrigerator temperature kept reliably for at least 72 h. The decay of pulps led to significant decreases in SSCs, firmness, pH, and L* and a* values. The mechanism underlying these changes could be inferred from the declined malate, citrate, uridine, UMP, and amino acid contents and the increased ethanol and lactate levels, which were attributed to the autologous metabolism and microbial activities of the genera *Enterobacteriaceae*, *Leuconostoc*, and *Lactococcus*. These findings cast new light on the quality changes of fresh-cut watermelons in common storage conditions, thereby providing novel clues for monitoring pulp quality and extending shelf life through targeted inhibition of metabolic and microbial activities in future work. Moreover, relevant information reported in this paper could substantially benefit the establishment of guidelines and standards involving fresh-cut watermelons.

## Figures and Tables

**Table 1:** Parameters of OPLS-DA models constructed between groups of fresh cubes and cubes that have been stored at 4 °C or 28 °C for 0, 3, 6, 12, 24, 36, 48 and 72 h.

OPLS-DA Models		Model Parameters			
Control Group	Treated Group	R2X	R2Y	Q2	P(CV-ANOVA)
Fresh	S3h_28	0.28	0.95	0.08	1.00
	S6h_28	0.36	0.91	0.03	0.99
	S12h_28	0.37	0.98	0.65	0.08
	S24h_28	0.39	0.94	0.54	0.20
	S36h_28	0.44	0.94	0.62	0.11
	S48h_28	0.44	0.95	0.80	0.01
	S72h_28	0.44	0.98	0.86	0.00
	S3h_4	0.37	0.89	−0.01	1.00
	S6h_4	0.35	0.94	0.38	0.43
	S12h_4	0.39	0.95	0.43	0.35
	S24h_4	0.38	0.92	−0.07	1.00
	S36h_4	0.33	0.91	−0.46	1.00
	S48h_4	0.34	0.96	0.25	0.68
	S72h_4	0.24	0.93	−0.54	1.00

## Data Availability

Not applicable.

## Supplementary Materials

The following supporting information can be downloaded at: https://www.mdpi.com/article/10.3390/foods11213423/s1, Figure S1: Typical 2D NMR spectra of watermelon extracts employed in ^1^H NMR signal assignment: (A) overlapping figure of ^1^H-^13^C HSQC, ^1^H-^13^C HMBC and ^1^H NMR; (B) overlapping figure of ^1^H-^1^H TOCSY and ^1^H NMR. Note: In figure A, ^1^H-^13^C HSQC signals are depicted in red and ^1^H-^13^C HMBC signals are depicted in blue; Figure S2: Permutation tests of OPLS-DA models constructed between groups of fresh cubes and cubes that have been stored at 4 °C or 28 °C for 0, 3, 6, 12, 24, 36, 48 and 72 h; Table S1: Color parameters of watermelon pulps stored at 4 °C or 28 °C for 0, 3, 6, 12, 24, 36, 48 and 72 h; Table S2: NMR signal assignment table of watermelon extracts.
