# Supplementary material for: Systematic Study of the Sensory Quality, Metabolomics, and Microbial Community of Fresh-Cut Watermelon Provides New Clues for Its Quality Control and Preservation
Source: Foods. 2022 Oct 28;11(21):3423. doi: 10.3390/foods11213423 (PMC9658764; doi:10.3390/foods11213423)
Supplement: Supplementary file 1 [file foods-11-03423-s001.zip › foods-1986833-supplementary.pdf]

Supplementary Information

Systematic Study of the Sensory Quality, Metabolomics, and Microbial Community of Fresh-Cut Watermelon Provides New Clues for Its Quality Control and Preservation

Yili Hu [1,2], Yi Cai [3], Haibin Wang [2], Yin Xiong [1], Xinyu Zhang [2], Liying Wei [2] and Zhixian Qiao [4,*]

[1] National R&D Center for Se-Rich Agricultural Products Processing, School of Modern Industry for Selenium Science and Engineering, Wuhan Polytechnic University, Wuhan 430023, China
[2] Hubei Key Laboratory for Processing and Transformation of Agricultural Products, School of Food Science and Engineering, Wuhan Polytechnic University, Wuhan 430023, China
[3] Tianjin Institute of Industrial Biotechnology, Chinese Academy of Sciences, Tianjin 300308, China
[4] Institute of Hydrobiology, Chinese Academy of Sciences, Wuhan 430072, China
* Correspondence: qzhxian@ihb.ac.cn

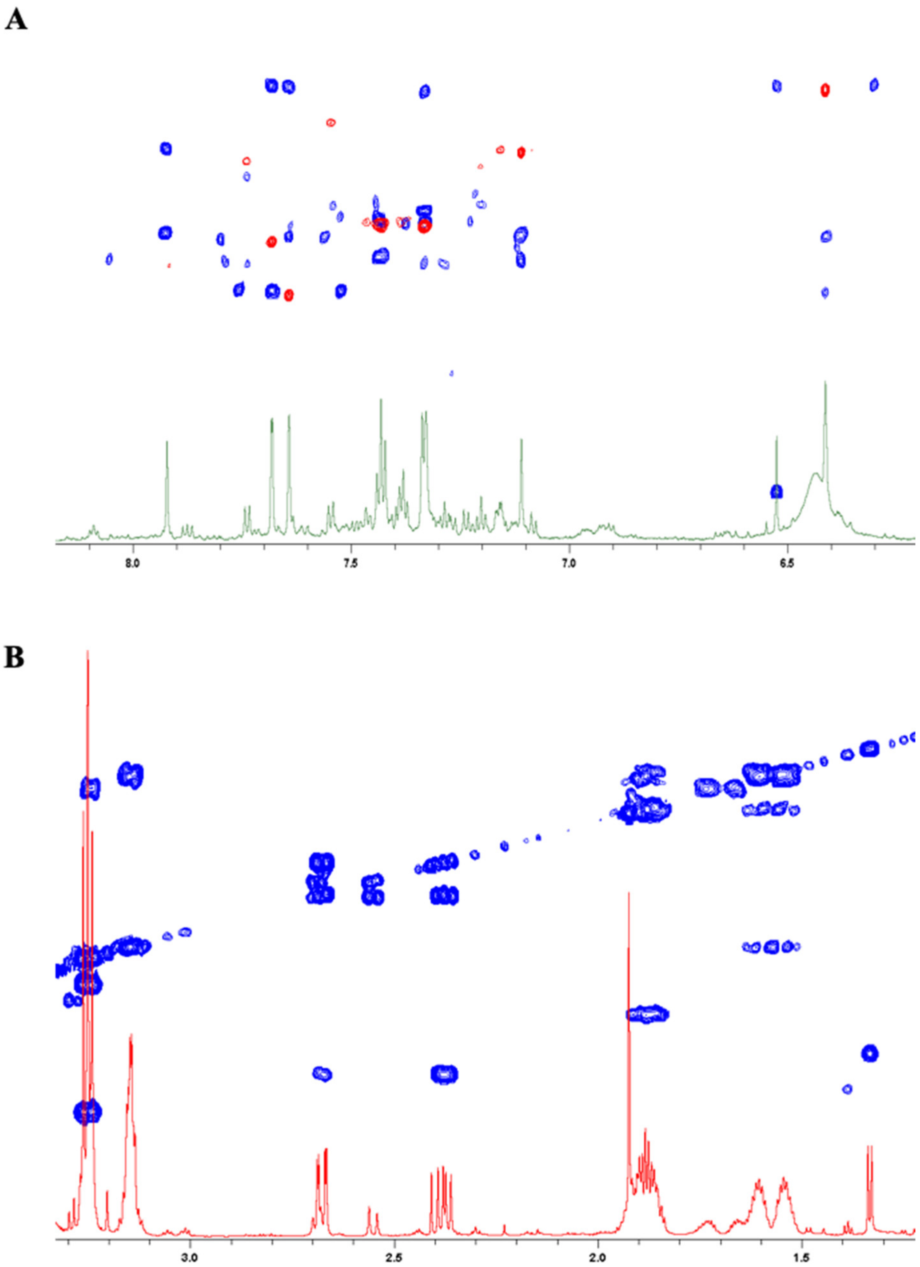

Figure S1. Typical 2D NMR spectra of watermelon extracts employed in ^{1}H NMR signal assignment: (A) overlapping figure of ^{1}H-^{13}C HSQC, ^{1}H-^{13}C HMBC and ^{1}H NMR; (B) overlapping figure of ^{1}H-^{1}H TOCSY and ^{1}H NMR. Note: In figure A, ^{1}H-^{13}C HSQC signals are depicted in red and ^{1}H-^{13}C HMBC signals are depicted in blue.

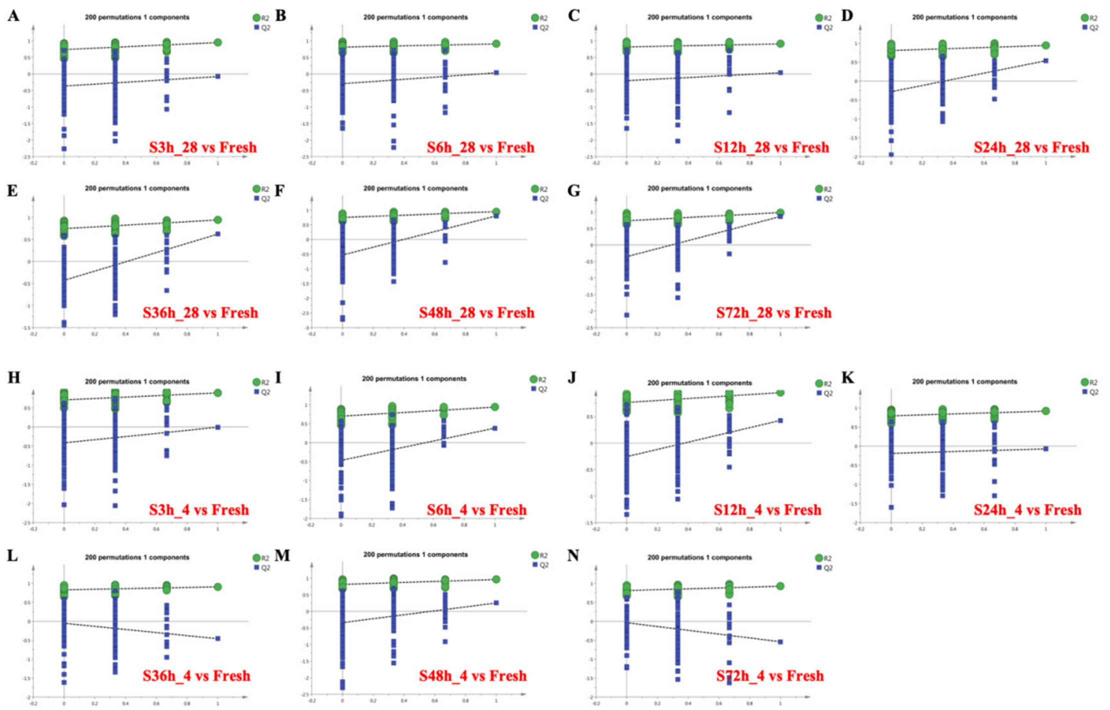

Figure S2. Permutation tests of OPLS-DA models constructed between groups of fresh cubes and cubes that have been stored at 4 °C or 28 °C for 0, 3, 6, 12, 24, 36, 48 and 72 h.

Table S1. Color parameters of watermelon pulps stored at 4 °C or 28 °C for 0, 3, 6, 12, 24, 36, 48 and 72 h.

Groups	Color parameters				
	L*	a	b	chroma	Hue
Fresh	40.5±1.9	28.1±1.7	11.3±1.2	30.3±2.1	22.0±2.0
S3h_28	42.6±2.2	27.5±0.9	10.9±1.5	29.6±1.8	21.5±2.6
S6h_28	41.3±1.8	27.2±3.4	11.8±1.2	29.7±3.6	23.7±3.6
S12h_28	40.5±3.4	27.1±3.3	11.4±1.1	29.4±3.4	23.0±1.5
S24h_28	40.1±2.3	27.6±4.3	11.0±1.6	29.7±4.6	22.0±3.3
S36h_28	38.1±4.6	28.6±3.7	10.8±1.6	30.5±4.1	20.8±3.5
S48h_28	32.3±3.6***	25.1±4.7	11.7±2.2	27.7±5.2	25.1±1.8*
S72h_28	27.5±2.1***	18.4±3.4***	11.9±1.0	21.9±3.5***	33.4±4.9***
S3h_4	43.0±14.3	27.4±4.7	11.5±2.0	29.7±5.1	22.8±2.1
S6h_4	41.8±8.9	27.2±2.0	10.8±0.5	29.2±2.1	21.7±1.5
S12h_4	39.2±2.6	29.7±3.0	11.1±2.0	31.7±3.5	20.7±4.0
S24h_4	40.3±3.7	27.4±2.5	10.8±1.1	29.4±2.7	21.6±1.7
S36h_4	39.4±2.8	27.2±2.3	11.1±0.7	29.4±2.4	22.2±2.3
S48h_4	37.7±2.2	28.3±4.3	11.7±1.1	30.6±4.4	22.6±2.0
S72h_4	38.6±3.1	28.1±3.8	11.2±2.3	30.2±4.4	21.8±4.1

Table S2. NMR signal assignment table of watermelon extracts.

Number	Metabolites	Groups	δ¹H	δ¹³C
1	isoleucine	δCH3	0.94 (t)	14.3
		βCH-CH3	1.01 (d)	17.7
		γCH2	1.27 (m)	27.0
		γCH2′	1.47 (m)	27.0
		βCH	1.98 (m)	38.9
		αCH	3.68 (d)	63.0
		COOH		177.0
2	leucine	δCH3	0.94 (d)	24.4
		δ′CH3	0.97 (d)	25.1
		βCH2	1.70 (m)	42.4
		γCH	1.72 (m)	26.5
		βCH2′	1.74 (m)	42.4
		αCH	3.74 (m)	56.5
		COOH		117.6
3	valine	γCH3	1.00 (d)	19.4
		γ′CH3	1.05 (d)	20.7
		βCH	2.28 (m)	31.5
		αCH	3.62 (d)	63.0
		COOH		177.4
4	ethanol	CH3	1.19 (t)	19.4
		CH2	3.66 (q)	60.1
5	lactate	CH3	1.33 (d)	22.8
		CH	4.11 (q)	71.3
		COOH		185.2
6	alanine	CH3	1.48 (d)	18.8
		CH	3.79 (m)	52.9
		COOH		178.5
7	citruline	γCH2	1.56 (m)	26.8
		βCH2	1.88 (m)	31.2
		δCH2	3.14 (m)	43.3
		αCH	3.71 (m)	57.9
		COOH		160.1
		CO		178.6
8	lysine	γCH2	1.45 (m)	24.6
		γCH2′	1.51 (m)	24.6
		δCH2	1.72 (m)	28.5
		βCH2	1.91 (m)	32.9
		εCH2	3.03 (t)	41.9
		CH	3.76	57.7
		COOH		177.5
9	glutamate	βCH2	2.07	29.7
		γCH2	2.36	36.0
		αCH	3.74	57.4
		1-COOH		177.0
		5-COOH		184.1
10	acetate	CH3	1.92 (s)	26.0
		COOH		nd
11	glutamine	βCH2	2.13 (m)	29.8
		γCH2	2.46 (m)	36.2
		αCH	3.78 (t)	57.9

		α-COOH		176.8
		COOH		180.5
12	4-aminobutyrate	βCH2	1.88 (m)	26.3
		αCH2	2.30 (t)	37.3
		γCH2	3.02 (t)	42.3
		COOH		184.1
13	malate	CH2	2.38 (dd)	45.2
		CH2	2.69 (dd)	45.2
		CH	4.32 (dd)	72.8
		1-COOH		183.1
		4-COOH		183.3
14	succinate	CH2	2.41 (s)	36.9
		COOH		185.1
15	citrate	CH2	2.55 (dd)	48.4
		CH2	2.68 (dd)	48.4
		C-OH		78.2
		1/5-COOH		181.7
		3'-COOH		184.4
16	aspartate	CH2	2.68 (dd)	37.1
		CH2	2.82 (dd)	37.1
		CH	3.91	52.9
17	choline	(CH3)3	3.21 (s)	56.7
		NCH2	3.52 (m)	70.1
		CH2OH	4.07 (m)	57.8
19	methanol	CH3	3.36 (s)	51.7
21	glycine	CH2	3.57 (s)	44.5
		COOH		175.1
23	fructose	1-CH	3.68 (d)	
		3-CH	4.12 (d)	77.8
		4-CH	4.01 (m)	
		5-CH	4.06 (m)	
		6-CH2	3.80 (m)	
18	β-glucose	1-CH	4.65 (d)	98.9
		4-CH	3.90	nd
		6-CH2	3.75	nd
		5-CH	3.69	nd
		3-CH	3.49	nd
		2-CH	3.26	nd
24	ascorbate	4-CH	4.85 (d)	nd
		5-CH	3.98 (m)	nd
		6-CH2	3.65 (d)	nd
20	α-glucose	1-CH	5.25 (d)	95.2
		5-CH	3.84	nd
		3-CH	3.73	75.2
		2-CH	3.57	nd
		4-CH	3.43	nd
		6-CH2	3.73	nd
22	sucrose	Glc-1-CH	5.42 (d)	95.2
		Fru-3-CH	4.22 (d)	79.4
		Fru-4-CH	3.89 (m)	81.4
		Fru-5-CH	3.84 (m)	72.4
		Glc-5-CH	3.83 (m)	81.4
		Glc-6-CH2	3.82 (m)	62.0

		Fru-6-CH2	3.81 (m)	60.1
		Glc-3-CH	3.76 (dd)	72.6
		Fru-1-CH2	3.69 (s)	64.3
		Glc-2-CH	3.56 (dd)	70.9
		Glc-4-CH	3.47 (dd)	69.1
25	uridine	CH2-OH	3.81	nd
		4-CH	4.12	nd
		3-CH	4.23	nd
		2-CH	4.34	76.9
		1-CH	5.92 (d)	92.3
		5-CH	5.91 (d)	105.5
		6-CH	7.88 (d)	144.5
		2-CO		154.9
		4-CO		168.9
26	UMP	CH2-OH	3.97	65.8
		4-CH	4.28	86.8
		3-CH	4.34	72.5
		2-CH	4.41	76.6
		1-CH	5.98 (d)	91.2
		5-CH	5.99 (d)	105.5
		6-CH	7.98 (d)	144.7
27	inosine	1-CH	6.06 (d)	90.9
		8-CH	8.26 (s)	148.9
		2-CH	8.36 (s)	142.7
28	ferulic acid	βCH	6.38 (d)	105.1
		αCH	7.35 (d)	nd
			7.08	
29	chlorogenic acid	βCH	6.41 (d)	nd
		γCH	7.66 (d)	nd
30	cis-AcoA	CH	6.48	nd
31	fumarate	CH	6.53 (s)	138.2
		COOH		177.2
32	4-HBA	3/5-CH	6.96 (d)	117.6
		2/6-CH	7.64 (d)	133.9
33	histidine	2-CH	7.10 (s)	120.2
		4-CH	7.90 (s)	139.1
		COOH		176.9
34	tryptophan	5-CH	7.15 (d)	nd
		6-CH	7.29 (d)	nd
		7-CH	7.54 (d)	nd
		4-CH	7.74 (d)	nd
35	phenylalanine	CH2	3.13 (dd)	39.5
		CH2′	3.28 (dd)	39.5
		N-CH	4.00 (dd)	58.8
		o-CH	7.33 (m)	132.0
		p-CH	7.38 (m)	130.9
		m-CH	7.43 (m)	132.1
		quaternary C		138.0
		COOH		175.9
36	tyrosine	CH2	3.06 (dd)	38.6
		CH2′	3.19 (dd)	38.6
		N-CH	3.94 (dd)	59.0
		o-CH to C-OH	6.90 (m)	118.3

		m-CH to C-OH	7.20 (m)	133.4
		quaternary C		129.6
		C-OH		158.0
		COOH		177.1
37	trigonelline	1-CH	9.13 (s)	148.0
		2/4-CH	8.84 (dd)	148.0
		3-CH	8.08 (d)	139.2
38	formate	HCOOH	8.46 (s)	172.4
